# Assessing the Impact of Intravenous and Parenteral Nutrition (IVPN) Network on the Development and Productivity of Pharmacists, Healthcare Providers, and Researchers

**DOI:** 10.7759/cureus.93583

**Published:** 2025-09-30

**Authors:** Muna Barakat, Nagham Sheblak, Kanika Vats, Sundus Shukar, Noor Breik, Ahmad El Ouweini, Faten Hamed, Reem Ahmed, Osama Tabbara

**Affiliations:** 1 Department of Clinical Pharmacy and Therapeutics, Faculty of Pharmacy, Applied Science Private University, Amman, JOR; 2 Department of Clinical Research Listserv, Intravenous and Parenteral Nutrition (IVPN) Network, Fujairah, ARE; 3 Department of Research and Development, Medical Agency for Research and Statistics, Riyadh, SAU; 4 Department of Research and Development, Emirates Classification Society (TASNEEF), Abu Dhabi, ARE; 5 Department of Management, School of Commerce and Management, Om Sterling Global University, Hisar, IND; 6 Department of Pharmacy Administration, School of Pharmacy, Xi'an Jiaotong University, Xi'an, CHN; 7 Department of Community Health Sciences, Rady Faculty of Health Sciences, University of Manitoba, Winnipeg, CAN; 8 Department of Pharmacy Practice, College of Pharmacy, Dubai Medical University, Dubai, ARE; 9 School of Pharmacy, Lebanese International University, Beirut, LBN; 10 Department of Clinical Pharmacy, Saudi German Hospital, Cairo, EGY

**Keywords:** healthcare professionals, ivpn network, knowledge sharing, patient outcomes, professional development

## Abstract

Background: Effective knowledge sharing and professional networking are critical for enhancing healthcare practice. The Intravenous and Parenteral Nutrition (IVPN) Network, originally focused on nutrition support, has expanded into a global platform supporting professional development across multiple disciplines. However, its broader impact on healthcare providers’ growth and practice remains underexplored. This study aimed to evaluate the perceived influence of the IVPN Network through four objectives: (1) to assess awareness and usage, (2) to identify training needs, (3) to evaluate its impact on professional development, collaboration, and patient care, and (4) to measure user satisfaction and advocacy.

Methods: A cross-sectional, web-based survey was conducted between September 2024 and January 2025 using non-probability snowball sampling. Eligible participants were pharmacists, healthcare providers, and researchers aged ≥18 years who had engaged with IVPN activities within the past two years. The questionnaire, developed from literature and validated by experts (Cronbach’s alpha ≥ 0.79), included demographic items and Likert-scale questions across the four study domains. Data were analyzed using SPSS version 26 (IBM Corp., Armonk, NY). Mann-Whitney U tests were used for sex-based comparisons, Pearson correlation for age and performance scores, and Kruskal-Wallis tests for training effects.

Results: A total of 493 healthcare professionals from 39 countries responded, with the majority from the Gulf region. Awareness of the IVPN Network was relatively high, although patterns of engagement varied. Formal or informal training was significantly associated with higher perceived benefits. Participants reported that the network enhanced access to clinical information, communication, and decision-making, and supported professional collaboration. Overall satisfaction was high, with 95.1% indicating they would recommend the platform.

Conclusion: The IVPN Network is perceived to support professional development and collaboration among healthcare providers across diverse regions. While findings provide valuable insights into members’ experiences, they reflect self-reported perceptions and cannot be interpreted as causal effects due to the cross-sectional design, reliance on subjective data, and absence of control groups. Future research should incorporate longitudinal designs, objective clinical outcome measures, and independent evaluations to confirm and extend these findings.

## Introduction

Since its inception in 2012, the Intravenous and Parenteral Nutrition (IVPN) Network has served as an innovative platform dedicated to fostering collaboration among healthcare professionals, researchers, and pharmacists specializing in intravenous and parenteral nutrition [1]. The primary aim was to create a space where practitioners could share knowledge, exchange experiences, and stay updated on emerging research in this complex field. Over the years, the network has expanded significantly, transforming from a basic discussion forum into a comprehensive resource supporting professional development and practice improvement on regional and global scales [1]. Since July 2020, the IVPN Network has expanded its scope by offering diverse services, including online educational programs, expert consultations, and specialized discussion groups, all aimed at enhancing the quality of nutrition care [1].

The network comprises 16 dedicated listservs that address a broad range of topics, including clinical research, pharmacogenomics, oncology, and other specialized areas [1]. These electronic forums facilitate rapid dissemination of the latest scientific findings, clinical techniques, and innovative approaches, encouraging interdisciplinary collaboration among healthcare providers. Additionally, the IVPN Network offers free webinars, online courses, and expert clinical assistance, resources designed to keep professionals current with evolving best practices, improve patient safety, and optimize clinical outcomes. Its diverse offerings exemplify a commitment to fostering a vibrant and engaged community of healthcare professionals dedicated to advancing the science and practice of intravenous and parenteral nutrition (IVPN).

Pharmacists, in particular, are central to ensuring safe and effective administration. Their specialized knowledge is vital in managing complex nutritional regimens, preventing adverse events, and tailoring treatments to meet individual patient needs [2]. Given the rapid pace of innovation in medical therapies, continuous professional education is essential for pharmacists to maintain competence and improve clinical practice. Lifelong learning initiatives bolster individual expertise and strengthen healthcare systems globally, especially in the context of evolving healthcare challenges [3]. Such ongoing development is critical in achieving the Sustainable Development Goals (SDGs), particularly Goal 3, which aims to ensure healthy lives and promote well-being. Within this framework, target 3.8 emphasizes the importance of universal health coverage, including access to quality medicines and health services, roles in which pharmacists are increasingly engaged [4].

Despite the importance of continuous learning, studies reveal that pharmacists encounter barriers when engaging with digital platforms for professional development. Research from North America indicates that although social media use among pharmacists and pharmacy students is widespread (82.8% reported using social media), active participation in professional activities on these platforms remains limited. Common barriers include time constraints (38.8%), concerns regarding legal liability (47.6%), and a perception that such engagement offers limited tangible benefits [5,6]. Similar patterns are observed internationally; for instance, in Saudi Arabia, younger pharmacists tend to leverage social media primarily for knowledge exchange and networking, illustrating its emerging role in professional growth and collaboration within different cultural contexts [7]. These findings highlight the opportunities and challenges of utilizing online platforms for continuing education and professional networking.

The IVPN Network promotes knowledge sharing and interdisciplinary collaboration among healthcare professionals in nutrition therapy. While its immediate benefits in communication and community building are recognized, its broader impact on improving professional skills, fostering research partnerships, and enhancing healthcare delivery remains understudied. Therefore, this study aims to evaluate the IVPN Network’s influence on healthcare professionals through four main objectives: (1) to assess awareness and usage of the IVPN Network; (2) to identify training needs for effective engagement; (3) to evaluate its impact on professional development, collaboration, and patient care; and (4) to measure user satisfaction and advocacy.

This article was first presented as a poster at the First Community Pharmacy Conference, held on April 26, 2025, in Dubai, United Arab Emirates, where it received second place in the poster presentation category.

## Materials and methods

Study design

A cross-sectional study was conducted between September 2024 and January 2025 to assess the impact of the IVPN Network on the professional development, collaboration, and efficiency of pharmacists, healthcare providers, and researchers. The study evaluated the influence of the IVPN Network on participants’ ability to access and utilize up-to-date pharmaceutical information, improve patient care outcomes, enhance decision-making processes, engage in research activities, and adopt new healthcare technologies.

Participants and sampling

Data were collected through a self-administered web-based questionnaire distributed to individuals who had attended IVPN events or were registered on an IVPN listserv. A total of 1,200 invitations were distributed through email, LinkedIn, and Facebook posts. Of these, 556 individuals initiated the survey, 493 provided complete responses (response rate = 41.1%), and 63 discontinued before completion. This strategy was chosen to facilitate broad participation across diverse healthcare settings. A non-probability snowball sampling approach was used to recruit participants. The study population included pharmacists, healthcare providers, and researchers affiliated with healthcare institutions and the IVPN community. Sample size was calculated using G*Power version 3.1 (Heinrich-Heine-Universität Düsseldorf, Düsseldorf, Germany) with the following parameters: medium effect size (Cohen’s d = 0.3), alpha = 0.05, power (1-β) = 0.95, and margin of error ±3%. This yielded a minimum required sample size of 385 participants.

Inclusion and exclusion criteria

The target population included pharmacists, healthcare professionals, and researchers affiliated with the IVPN community. Inclusion criteria were as follows: (1) being ≥18 years of age; (2) participation in IVPN webinars, events, or resource use within the last two years; and (3) provision of informed consent. Exclusion criteria were as follows: (1) under 18 years of age or (2) no documented or self-reported engagement with IVPN Network activities in the last two years.

Study tool and data collection

Recruitment was carried out via professional networks, LinkedIn groups, the official IVPN Facebook page, and direct email invitations sent to members of the IVPN listserv. To enhance participation, three reminder emails were issued at two-week intervals, using identical wording to the initial invitation to minimize response bias. Participants were also encouraged to share the survey link within their professional circles, thereby broadening outreach and maximizing response rates.

**Figure 1:**
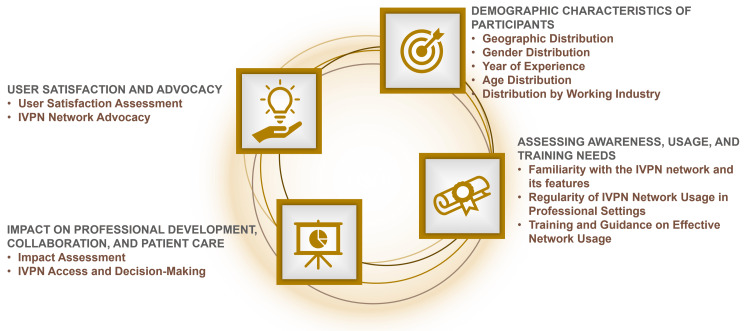
Main Themes of the Study Questionnaire IVPN: intravenous and parenteral nutrition

The questionnaire included an online consent form, ensuring voluntary and anonymous participation. The Google Forms "Required" option was enabled to prevent incomplete responses. The questionnaire was developed following an extensive literature review [8-11] and initially drafted by two authors. It was then pilot-tested by the remaining seven authors to ensure clarity, consistency, and content validity. Validated scales assessing professional development, knowledge exchange, and productivity in healthcare networking platforms were incorporated [12,13]. The final study questionnaire was structured around four domains aligned with the study objectives, as illustrated in Figure 1.

These domains include (1) demographic characteristics of the participants; (2) assessment of awareness, usage, and training needs; (3) evaluation of the impact on professional development, collaboration, and patient care; and (4) user satisfaction and advocacy. The complete questionnaire used in this study is provided in the Appendices.

Responses were measured using a 5-point Likert scale (1 = strongly disagree, 5 = strongly agree) to ensure scalability and response variability. Negative items were reverse-coded to minimize response bias. To establish content validity, the questionnaire was evaluated by a panel of eight experts specializing in healthcare, pharmacy education, and professional networking. Reliability was assessed using Cronbach’s alpha, with an internal consistency coefficient of ≥0.79, indicating an acceptable level of reliability.

Ethics and transparency statement

The study adhered to the ethical principles outlined in the Declaration of Helsinki. Prior to commencing data collection, approval was secured from the Institutional Review Board (IRB) of Applied Science Private University (approval number: 2024-PHA-36). Participation was entirely voluntary and anonymous, with no personal identifiers collected.

Statistical analyses

Statistical analyses were conducted using SPSS version 26 (IBM Corp., Armonk, NY) to evaluate the impact of various factors on the IVPN Network’s performance. The demographic characteristics of the participants were summarized, and several statistical tests were applied to assess the relationships between variables.

Data were screened for completeness, and only fully completed responses were included in the analysis; no imputation was performed for missing values. As the Shapiro-Wilk test indicated non-normal distribution of performance scores, non-parametric tests were applied. A Mann-Whitney U test compared performance scores between male and female participants (independent variable: sex; dependent variable: total performance score). A Pearson correlation examined associations between age (continuous variable) and performance score (continuous variable). A Kruskal-Wallis test compared performance across training groups (independent variable: training, category: none, informal, and formal; dependent variable: performance score).

Bias mitigation measures

Although this study focused on the IVPN Network, the authors’ direct involvement was limited to a single listserv out of the 16 that comprise the network. The survey was distributed to all eligible members, providing a broad opportunity for participation and reducing the risk of intentional selection bias. While snowball sampling was used, recruitment was conducted through multiple channels, including professional networks, LinkedIn groups, the IVPN Facebook page, and direct email invitations, ensuring diverse initial outreach and further minimizing selection bias.

Participation was voluntary and anonymous, with responses collected using standardized, validated Likert scales. To mitigate bias in interpretation, data analysis and outcome validation were conducted independently by all authors, followed by a collective review; any discrepancies were resolved through discussion and mutual consensus. Findings are reported transparently, highlighting both strengths and areas for improvement, and emphasizing perceived impacts rather than objective outcomes.

Additionally, the questionnaire was developed from an extensive literature review and incorporated validated scales assessing professional development, knowledge exchange, and productivity. Drafted by two authors and pilot-tested by the remaining seven, the study questionnaire was refined for clarity and consistency. This process reduced the risk of measurement error, ensured alignment with study objectives, and enhanced the validity of the findings.

## Results

Our study results reflect a broad age range of respondents, from 19 to 68 years, with a mean age of 38.3 years and a standard deviation (SD) of 10.3, suggesting a moderate spread. This distribution highlights a diverse cohort comprising early-career, mid-career, and experienced professionals, offering a broad spectrum of professional backgrounds and perspectives.

The study initially included 556 respondents, of whom 493 agreed to participate in the questionnaire, while the remaining individuals opted not to proceed further.

Demographic characteristics of the participants

This section provides an overview of the demographic profile of the respondents, offering context for interpreting subsequent findings on awareness, usage, and professional outcomes. Demographic characteristics such as age, gender, years of experience, professional affiliation, and geographical distribution are presented in Table 1.

**Table 1:** Demographic Characteristics of the Study Participants (N = 493) *Residential countries: Gulf countries include Bahrain, Kuwait, Oman, Qatar, Saudi Arabia, and the United Arab Emirates. Asian countries include Afghanistan, Bangladesh, China, India, Indonesia, Pakistan, the Philippines, Sri Lanka, Kyrgyzstan, and Uzbekistan. Middle East countries include Iraq, Jordan, Lebanon, Palestine, Syria, and Yemen, and exclude the Gulf, Egypt, and Libya. North African countries include Algeria, Egypt, Libya, Morocco, and Sudan. Sub-Saharan African countries include Ghana, Nigeria, Rwanda, and Somalia. European countries include Germany, Romania, Russia, Serbia and Montenegro, Spain, and Turkey (partly in Europe). North American countries include Canada and the United States. SD: standard deviation

Variable	Number	%
Gender		
Male	257	52.1%
Female	236	47.9%
Years of experience		
0-5 years	119	24.1%
6-10 years	97	19.7%
>10 years	277	56.2%
Major		
Academia	37	7.5%
Community pharmacies	48	9.7%
Hospitals	272	55.2%
Industries	4	0.8%
Others	56	11.4%
Pharmaceutical companies	12	2.4%
Private clinics	57	11.6%
Research institutes	7	1.4%
Residential country*		
Gulf countries	340	69%
Middle East (excluding Gulf, Egypt, and Libya)	43	8.7%
North Africa	39	7.9%
Sub-Saharan Africa	8	1.6%
Asia	50	10.1%
Europe	8	1.6%
North America	5	1%
Age (years)	Mean	SD
	38.3	10.3

**Figure 2:**
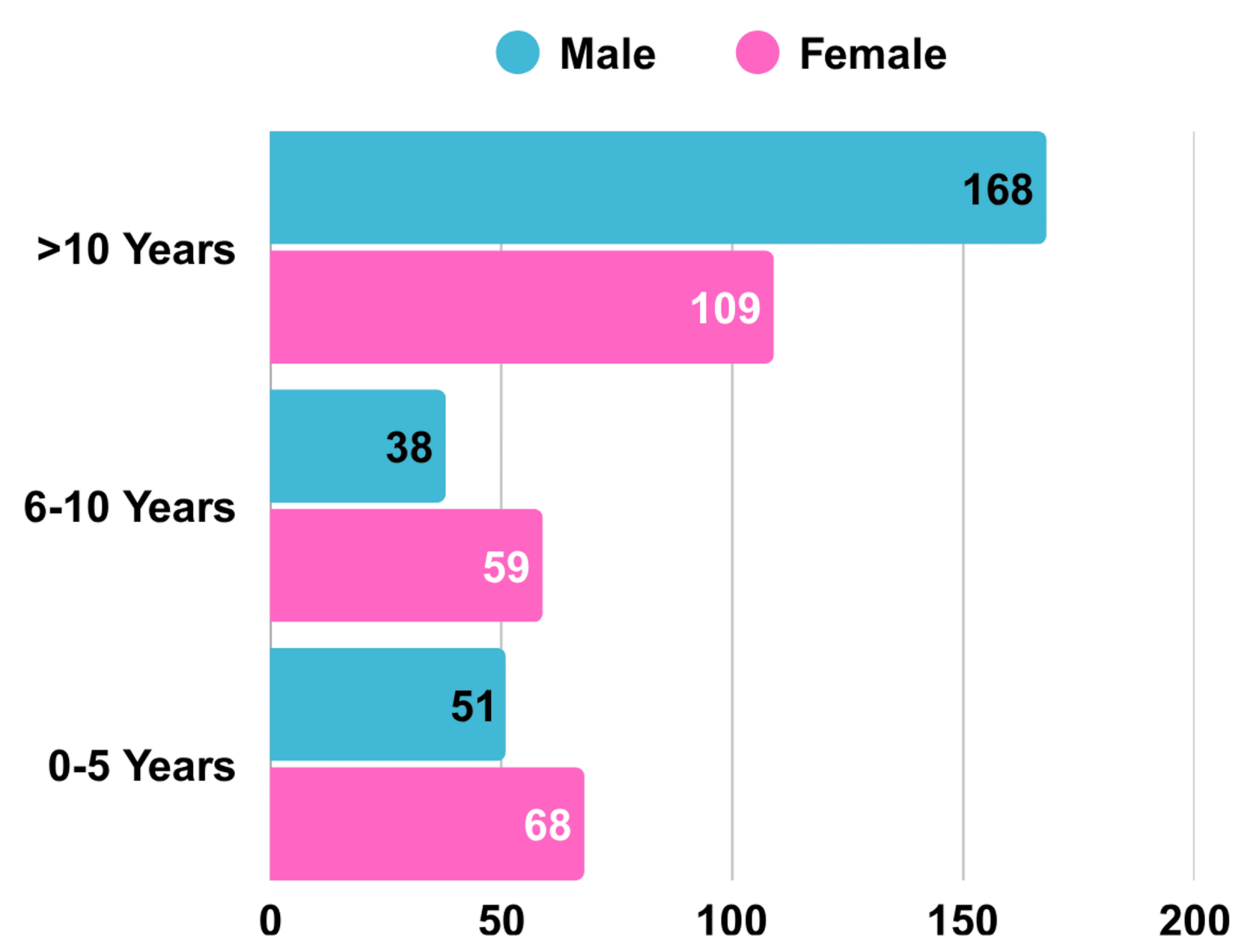
Gender Distribution by Years of Experience

Gender distribution shows that there were 257 (52.1%) male participants and 236 (47.9%) female participants, as shown in Figure 2. For the year of experience responses, female participants have a more evenly distributed range of experience levels, with the highest proportion (46.2%) falling into the >10 years category. However, there is a notable representation in the 0-5 years and 6-10 years categories, with 28.8% of female participants having 0-5 years of experience and 25% having 6-10 years. Male participants have more overall experience, with the largest proportion (65.4%) having more than 10 years of experience. Male participants also have a relatively small representation in the lower experience categories, with 19.8% having 0-5 years of experience and 14.8% having 6-10 years of experience.

The results indicate that male participants are more inclined to engage with the IVPN Network, especially in the >10 years’ experience category. Meanwhile, female participants appear to engage with the IVPN Network more during the early stages of their careers (0-5 years) and those with moderate experience (6-10 years). This could imply varying career progression trends or opportunities based on gender, which may merit further investigation.

**Figure 3:**
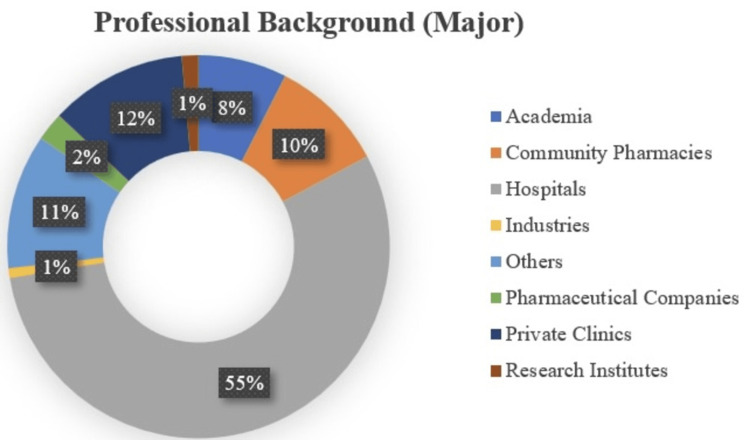
Distribution of Survey Responders Across Various Working Industries

Figure 3 shows the professional affiliations of the study participants. It highlights a strong focus on healthcare, particularly in hospitals and private clinics, with a smaller but relevant presence from academia, community pharmacies, and other sectors. The relatively low representation from industries and pharmaceutical companies suggests that the current services of the IVPN Network are more relevant to those working directly in healthcare services rather than in the pharmaceutical manufacturing or industrial sectors. It also highlights the prevalence of respondents from healthcare-related sectors, particularly hospitals and private clinics, while showing smaller representations from academia, community pharmacies, pharmaceutical companies, and other industries.

**Figure 4:**
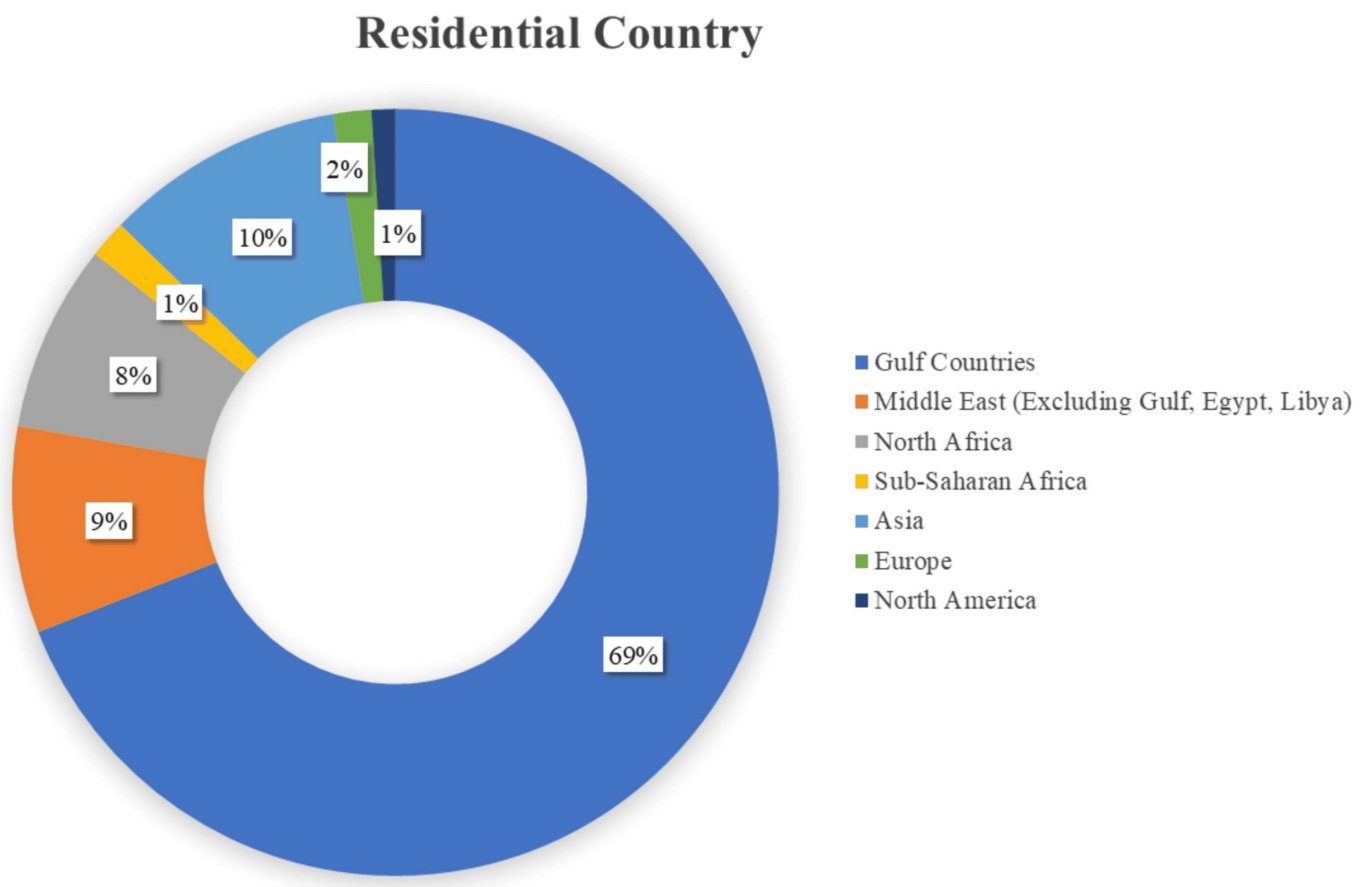
Global Distribution of Respondents by Country Gulf countries: Saudi Arabia, United Arab Emirates, Kuwait, Qatar, Bahrain, and Oman Middle East (excluding Gulf, Egypt, and Libya): Jordan, Lebanon, Syria, Palestine, Iraq, and Yemen North Africa: Egypt, Libya, Sudan, Algeria, Tunisia, and Morocco Sub-Saharan Africa: Nigeria, Kenya, Ghana, Ethiopia, South Africa, and other Sub-Saharan countries Asia: India, Pakistan, Bangladesh, the Philippines, Malaysia, and other Asian countries Europe: United Kingdom, Germany, France, Italy, Spain, and other European countries

The geographical distribution of the participants, as demonstrated in Figure 4, reflects a broad geographical distribution of responders (N = 493) spanning 39 countries and highlights the global reach of the IVPN Network. The results show a significant representation from the Gulf region (n = 340, 69%), with Saudi Arabia being the leading contributor (n = 301, 61.1%). Additionally, there is notable participation from Asia (n = 50, 10.1%), followed by respondents from the other Middle Eastern countries (n = 43, 8.7%). African countries (North and Sub-Saharan) have lower representation, while Europe and North America contributed minimally.

This geographic distribution suggests that the study findings will most likely reflect Gulf-based perspectives, possibly due to targeted outreach, professional networks, or regional healthcare collaboration, with some insights from Asia and the Middle East, suggesting potential gaps in awareness or engagement within those regions.

Assessment of the awareness, usage, and training needs

This section examines participants’ familiarity with the IVPN Network, their frequency of use, and the extent of training or guidance received. By exploring these elements, the results provide insight into how well the platform has penetrated professional practice, what gaps exist in user training, and how these factors may shape engagement with the network’s resources.

Familiarity With the IVPN Network and Its Features

As presented in Table 2, a majority of the respondents (67.7%) are somewhat familiar with the IVPN Network, with 36.9% being “very familiar” and 30.8% “familiar (naturally).” This suggests that IVPN Network has effectively reached and engaged its audience, ensuring users are well-informed about its features. The combined 32.3% (6.9% “not familiar” + 25.4% “somewhat familiar”) represents an opportunity for the IVPN Network to increase awareness and knowledge through targeted educational campaigns, user guides, or interactive tutorials.

**Table 2:** Familiarity, Usage, and Training on the IVPN Network Among Respondents IVPN: intravenous and parenteral nutrition

Questions	Number	(%)
How familiar are you with the IVPN Network and its features?		
Very familiar	182	36.9%
Familiar (naturally)	152	30.8%
Somewhat familiar	125	25.4%
Not familiar	34	6.9%
How often do you use the IVPN Network in your work?		
Daily	57	11.6%
Weekly	111	22.5%
Monthly	79	16%
Once in a while	176	35.7%
Never	70	14.2%
Have you received training or guidance on using the network effectively before?		
Yes, formal training	131	26.6%
Yes, informal guidance	131	26.6%
No	231	46.9%

Regularity of IVPN Network Usage in Professional Settings

As shown in Table 2, the majority (49.9%) either use IVPN resources infrequently or not at all, suggesting possible gaps in necessity, awareness, or accessibility. Of the respondents, 16% were moderate users who used the network monthly, suggesting periodic necessity rather than routine use, and 34.1% use the IVPN Network regularly (daily and weekly), indicating that it is a valuable asset in supporting and addressing the regular clinical needs of healthcare professionals.

Training and Guidance on Effective Network Usage

**Figure 5:**
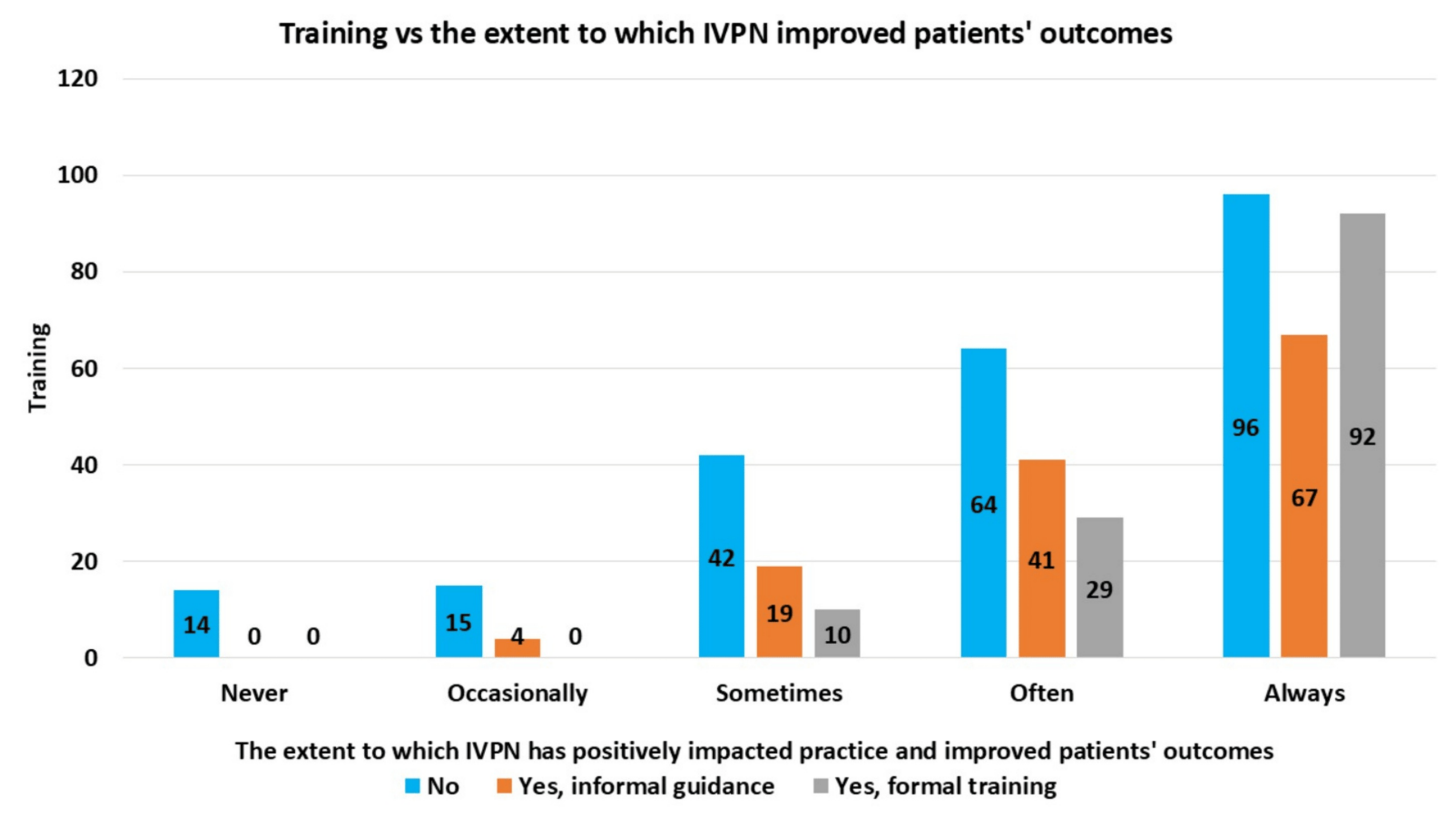
Association Between Training on the IVPN Network and Perceived Impact on Practice and Patient Outcomes IVPN: intravenous and parenteral nutrition

As demonstrated in Figure 5, nearly half of the respondents (46.9%) have not received any training or guidance on using the IVPN Network effectively. Of the respondents, 26.6% have received formal training, and 26.6% have received informal guidance. This suggests a varied approach to the IVPN Network training, with a notable number of individuals lacking structured support for using it effectively. The Kruskal-Wallis test showed that performance scores differed significantly among the three training groups: no training, informal guidance, and formal training (p = 0.001). Therefore, we conclude that the level of training significantly influences performance in using the IVPN Network to improve patient outcomes and medication safety.

Evaluation of the impact on professional development, collaboration, and patient care

Here, we assess the perceived impact of IVPN Network participation on various aspects of professional practice. The findings highlight whether respondents reported improvements in knowledge sharing, communication, decision-making, and patient care.

Based on the Likert scale (1 to 5), results show that most respondents strongly agree that the IVPN Network has positively impacted various aspects of their professional practice. Most notably, 52.3% of the respondents reported that IVPN "Always" improved their access to medical information. At the same time, similar positive responses were seen in areas such as professional communication, decision-making, and medication management. The mean score of 4.20 (SD = 0.92) confirms the network’s overall positive influence on healthcare professionals’ work and development. Table 3 presents the responses regarding how much the IVPN Network has contributed to various aspects of professional practice and development among healthcare professionals.

**Table 3:** Impact of IVPN Network on Professional Practice and Development IVPN: intravenous and parenteral nutrition

Statement	Number (%)				
	Never	Occasionally	Sometimes	Often	Always
To what extent do you agree with the following statements regarding the IVPN Network?					
IVPN has improved my access to up-to-date medical information and resources.	10 (2%)	18 (3.7%)	80 (16.2%)	127 (25.8%)	258 (52.3%)
IVPN has enhanced my professional communication skills.	10 (2%)	22 (4.5%)	68 (13.8%)	137 (27.8%)	256 (51.9%)
IVPN has helped me identify the areas of strength and weakness in my professional development.	13 (2.6%)	19 (3.9%)	80 (16.2%)	132 (26.8%)	249 (50.5%)
IVPN has influenced my decision-making process regarding pharmaceutical interventions.	15 (3%)	21 (4.3%)	82 (16.6%)	137 (27.8%)	238 (48.3%)
IVPN has enriched my collaboration and communication with other pharmacists, healthcare professionals, and researchers.	13 (2.6%)	18 (3.7%)	78 (15.8%)	135 (27.4%)	249 (50.5%)
IVPN has improved my skills in managing patient medication profiles and histories.	13 (2.6%)	22 (4.5%)	73 (14.8%)	132 (26.8%)	253 (51.3%)
IVPN has positively impacted my practice and reflected in improving my patients’ outcomes or safety regarding medication management	14 (2.8%)	19 (3.9%)	71 (14.4%)	134 (27.2%)	255 (51.7%)
IVPN has strengthened my ability to access and share medical information and resources.	9 (1.8%)	22 (4.5%)	69 (14%)	143 (29%)	250 (50.7%)
IVPN has helped me to encounter the practice daily-challenges properly.	16 (3.2%)	19 (3.9%)	76 (15.4%)	141 (28.6%)	241 (48.9%)

User satisfaction and advocacy

This section presents participants’ satisfaction with the IVPN Network’s resources and technical support, along with their willingness to recommend the platform to others. These measures serve as indicators of overall user experience and perceived value.

**Figure 6:**
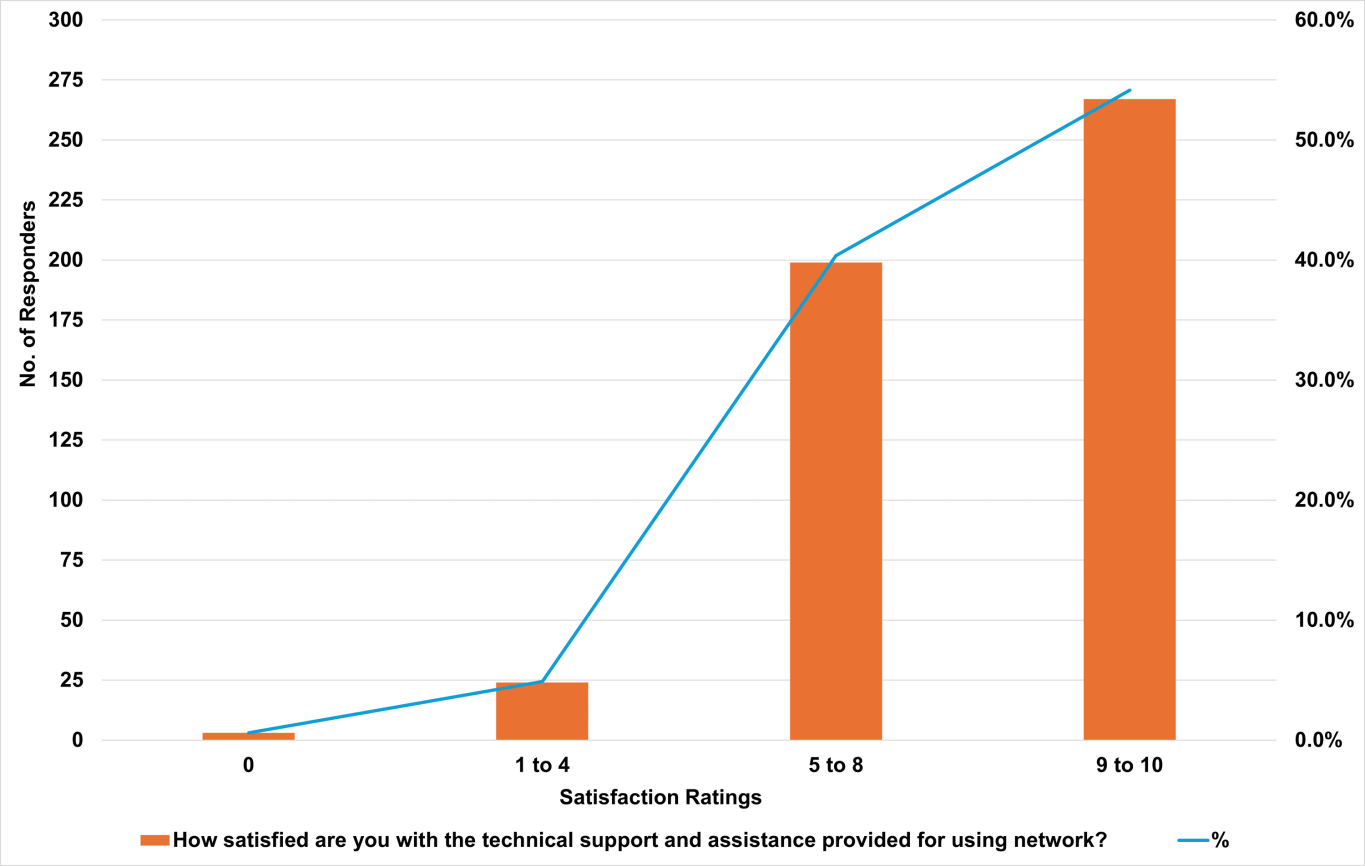
Satisfaction Levels With IVPN Network Technical Support IVPN: intravenous and parenteral nutrition

The study participants’ responses indicate they were overall satisfied with the technical support and assistance provided by IVPN Network, with an average satisfaction score of 8.27% out of 10. Furthermore, 54.2% rated their satisfaction between 9 and 10, reflecting a high level of contentment with the service. Additionally, 40.4% gave scores between 5 and 8, indicating moderate satisfaction. Only a small fraction (5.5%) expressed dissatisfaction. Of these, 4.9% rated their satisfaction between 1 and 4, and just 0.6% gave a rating of 0, indicating that a minimal number of users were unsatisfied with the technical support. Figure 6 illustrates the distribution of satisfaction ratings for the technical support and assistance provided for using the IVPN Network, highlighting the overall positive response from users.

**Figure 7:**
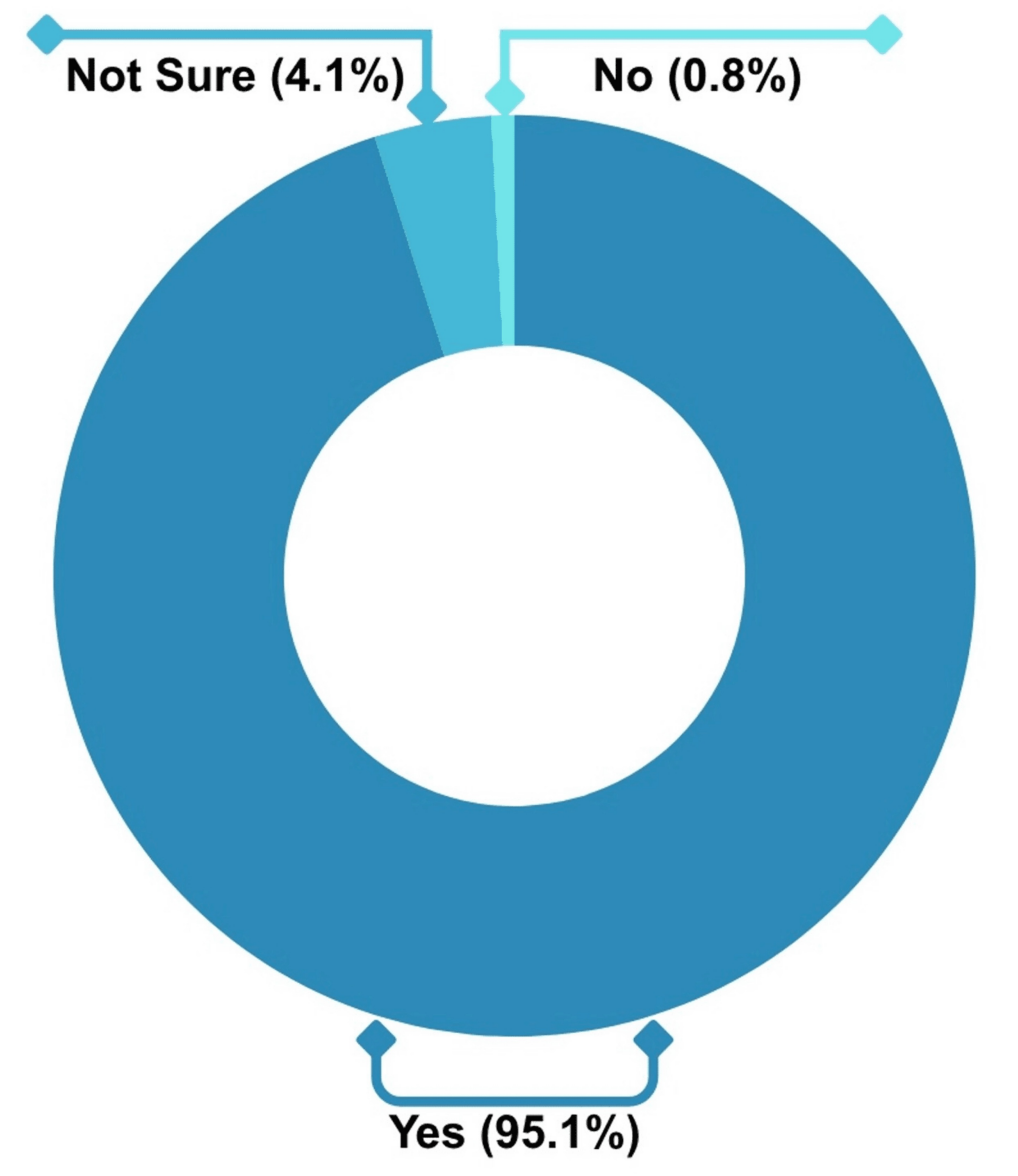
Likelihood of Recommending IVPN Network to Other Healthcare Professionals IVPN: intravenous and parenteral nutrition

Furthermore, the results demonstrate a strong positive sentiment toward the IVPN Network, with 95.1% of the respondents indicating that they would recommend it to other pharmacists or healthcare professionals. A very small percentage (0.8%) expressed that they would not recommend the IVPN Network, while 4.1% were unsure. This suggests that most users view the IVPN Network favorably, and its value is recognized within the healthcare community. However, a small group of users may have reservations or uncertainty about its broader application. Figure 7 shows healthcare professionals’ responses regarding their willingness to recommend the IVPN Network, showcasing users’ high level of endorsement.

The study results suggest that participants reported relatively high awareness of the IVPN Network, identified varied training needs, and perceived improvements in access to information, communication, decision-making, and professional collaboration. Furthermore, high satisfaction scores and strong willingness to recommend the network reflect positive user advocacy. These findings are aligned with the four study objectives and should be interpreted as participants’ perceptions rather than causal effects, given the cross-sectional and self-reported design.

## Discussion

Interprofessional collaboration and knowledge sharing are vital for improving patient care [14,15]. Pharmacists, as accessible providers, contribute significantly through clinical services, education, and research [16,17]. The IVPN Network was established to support collaborative learning and professional growth among pharmacists and healthcare professionals [1]. The present study provides valuable insights into the engagement, perceptions, and impact of the IVPN Network among healthcare professionals across diverse regions, primarily within the Gulf Cooperation Council countries. The demographic profile reveals a broad age range and a balanced gender distribution, with a notable trend of male respondents having greater experience (>10 years), particularly in engaging with the network. This aligns with existing literature suggesting that experienced clinicians often leverage digital platforms more extensively for continuous professional development and clinical support [18]. Conversely, the higher engagement of female respondents with early- to mid-career stages may reflect their pursuit of accessible, flexible learning resources to support evolving clinical roles.

The geographical distribution underscores the network’s significant reach within the Gulf region, especially in Saudi Arabia, with expanding participation from other Middle Eastern and Asian countries. This regional concentration may be driven by targeted outreach, regional collaborations, and the strategic positioning of healthcare institutions in these areas [19]. However, the relatively lower participation from Europe and North America suggests opportunities for the IVPN Network to broaden its global footprint, potentially through tailored content and international partnerships.

Regarding awareness and usage, approximately two-thirds of the respondents reported familiarity with the IVPN Network, with nearly half using it infrequently. This indicates that while the platform has established initial awareness, ongoing efforts are needed to promote consistent utilization. The importance of training emerged as a significant factor influencing effective use; participants who received formal or informal guidance performed better in leveraging the network’s features. This finding corroborates prior research emphasizing that structured training enhances digital literacy and optimizes the benefits of online professional communities [20,21]. Comprehensive onboarding programs and user support mechanisms can enhance engagement and clinical integration [20]. Participants also reported enhanced confidence, critical thinking, and professional growth, consistent with other studies emphasizing reflective, collaborative learning [22-24]. Improved communication, broader clinical perspectives, and global peer connections were noted, fostering reflective practice and reasoning [25-27]. While a small portion (5.5%) reported dissatisfaction or technical issues, such barriers are common in continuing professional development (CPD) settings [28]. Digital technologies have been pivotal during the COVID-19 pandemic, enabling remote healthcare and virtual learning, improving access while also revealing significant digital disparities [29,30].

The impact of the IVPN Network on professional development and patient care was generally perceived as positive. Over half of the respondents reported that the platform consistently improved their access to medical information, facilitated better communication, and supported clinical decision-making. Such findings are consistent with studies demonstrating that digital platforms enhance evidence-based practice by providing timely, peer-reviewed information, thereby reducing clinical uncertainty [31,32].

User satisfaction metrics further reinforce the platform’s effectiveness. The high satisfaction scores and the willingness of 95.1% of the participants to recommend the IVPN Network mirror results from prior evaluations of online professional communities, which highlight the importance of user-centered design, technical support, and relevant content in sustaining engagement [33,34]. The minimal dissatisfaction reported suggests that the platform largely meets user expectations; however, addressing the small fraction of dissatisfied users through targeted improvements could further enhance user experience.

Despite these strengths, the study uncovers several growth opportunities. The infrequent usage reported by many participants indicates barriers such as time constraints, lack of structured training, or limited institutional support. Addressing these issues through institutional integration, incentivization, and continuous education could foster more routine engagement [35]. Expanding outreach to underrepresented regions and sectors, including academia and industry, may diversify perspectives and enhance the platform’s relevance.

This study has several strengths. First, it addresses a timely and relevant topic, given the growing role of digital platforms in professional development and healthcare collaboration. Second, the focus on the IVPN Network provides a well-defined scope, offering a concrete case study of how specialized professional communities can influence professional growth, decision-making, and patient care. Third, the inclusion of participants from multiple countries enhances the diversity and generalizability of the findings, reflecting perspectives from various healthcare systems. Fourth, the use of a validated questionnaire, developed from literature and reviewed by experts with high internal consistency (Cronbach’s alpha ≥ 0.79), supports the reliability of the results. Finally, the study followed a structured methodology, with a clearly articulated design, inclusion criteria, sampling methods, and recruitment channels, which strengthens its reproducibility.

Limitations

This study has several important limitations that warrant consideration. First, while a conflict of interest was disclosed, several authors are affiliated with the IVPN Network. Although steps were taken to minimize bias, such as restricting direct involvement to a single listserv, ensuring anonymous participation, and conducting independent validation, the potential influence of this affiliation cannot be fully excluded. Second, the use of a non-probability snowball sampling method introduces the risk of selection bias, as participants recruited through professional networks may have been more likely to hold favorable perceptions of the IVPN Network. Third, the study relied exclusively on self-reported data, which are inherently vulnerable to social desirability bias and may not fully reflect actual behavioral changes or clinical outcomes. Fourth, the cross-sectional design and the absence of a control group prevent causal inference and make it difficult to isolate the effects of the IVPN Network from other professional development initiatives. In addition, the regional imbalance in the sample, with 69% of respondents from the Gulf region, limits the generalizability of findings to a truly global context. This concentration suggests that results may reflect region-specific practices or cultural factors rather than universal patterns. Furthermore, although the complete questionnaire is included in the Appendices, earlier reporting only provided partial coverage of Likert domains, which may have constrained replicability and transparency. Collectively, these limitations indicate that while the study provides valuable exploratory insights into the perceived impact of the IVPN Network, future research using probability-based sampling, broader geographical representation, longitudinal designs, and objective clinical outcome measures will be necessary to strengthen the evidence base.

## Conclusions

This study highlights that the IVPN Network is perceived to support professional development, collaboration, and practice productivity among pharmacists, healthcare providers, and researchers. Participants reported enhanced access to medical information, improved communication and decision-making, and greater confidence in their professional roles. High satisfaction rates and strong willingness to recommend the network further underscore its perceived value.

However, these findings are based on self-reported perceptions and should be interpreted with caution. The predominance of respondents from the Gulf region (69%) limits the global generalizability of the results, as they may reflect region-specific experiences rather than worldwide trends. In addition, the cross-sectional design and absence of objective outcome measures prevent causal conclusions. Future research should therefore incorporate broader geographical representation, comparison groups, and longitudinal designs with clinical outcome metrics to more rigorously evaluate the impact of the IVPN Network on healthcare practice.

## Appendices

Assessing IVPN Network’s impact on the development and productivity of pharmacists, healthcare providers, and researchers

This study is designed to assess the IVPN Network’s impact on the development and productivity of pharmacists, healthcare providers, and researchers. The survey is intended for all pharmacists, healthcare providers, and researchers who are registered in the IVPN Network and/or have participated in at least one IVPN Network session. Participation in this study will take five minutes only and is optional, and the data are anonymized. In addition, the confidentiality of the individual results is guaranteed in all circumstances.

Part 1: Sociodemographic data

Gender: (Male, Female)

Age (years): __________

Years of experience: (0-5 years, 6-10 years, >10 years)

Major: (Community pharmacies, Hospitals, Private clinics, Academia, Pharmaceutical companies, Industries, Research institutes, Others)

Residential country: __________

Part 2: Experience and perception toward the IVPN Network

How familiar are you with the IVPN Network and its features? (Very familiar, Familiar (naturally), Somewhat familiar, Not familiar)

How often do you use the IVPN Network in your work? (Never, Once in a while, Daily, Weekly, Monthly)

Have you received training or guidance on using the network effectively before? (Yes, formal training, Yes, informal guidance, No)

To what extent do you agree with the following statements regarding the IVPN Network (Always, Often, Sometimes, Occasionally, Never)

IVPN has improved my access to up-to-date medical information and resources.

IVPN has enhanced my professional communication skills.

IVPN has helped me identify the areas of strength and weakness in my professional development.

IVPN has influenced my decision-making process regarding pharmaceutical interventions.

IVPN has enriched my collaboration and communication with other pharmacists, healthcare professionals, and researchers.

IVPN has improved my skills in managing patient medication profiles and histories.

*IVPN has positively impacted my practice and reflected in improving my patients*’* outcomes or safety regarding medication management.*

IVPN has strengthened my ability to access and share medical information and resources.

IVPN has helped me to encounter the practice daily-challenges properly.

How satisfied are you with the technical support and assistance provided for using the network?

Not satisfied at all (0,1,2,3,4,5,6,7,8,9,10) Extremely satisfied

Based on your experience, would you recommend the IVPN Network to other pharmacists or healthcare professionals? (Yes, No, Not sure)
